# Parent preferences regarding stimulant therapies for ADHD: a comparison across six European countries

**DOI:** 10.1007/s00787-013-0515-6

**Published:** 2014-01-18

**Authors:** Beenish Nafees, Juliana Setyawan, Andrew Lloyd, Shehzad Ali, Sarah Hearn, Rahul Sasane, Edmund Sonuga-Barke, Paul Hodgkins

**Affiliations:** 1 Patient Reported Outcomes, Oxford Outcomes Ltd, Seacourt Tower, West Way, Oxford, OX2 0JJ UK; 2 Global Health Economics, Outcomes Research and Epidemiology, Shire, Wayne, PA USA; 3 Institute for Disorder of Impulse and Attention, School of Psychology, University of Southampton, Southampton, UK; 4 Department of Experimental Clinical and Health Psychology, Ghent University, Ghent, Belgium; 5 Present Address: Health Economics, Novartis Pharmaceuticals, East Hanover, NJ USA; 6 Nafees Consulting Limited, Communications House, 26 York Street, London, W1U 6PZ UK; 7 Present Address: Health Economics and Outcomes Research, Vertex Pharmaceuticals, Cambridge, MA USA

**Keywords:** ADHD, Patient preference, Discrete choice experiment, Stimulant therapy

## Abstract

The objective is to identify attributes of ADHD stimulant medications that influence treatment preferences of parents of children and adolescents with ADHD across six European countries, using a discrete choice experiment (DCE). Different attributes (and associated levels) of stimulant therapies were identified through literature review and clinician input. Attributes included duration and degree of symptom control after each dose, frequency of medication dosing, potential for treatment to be abused, the side effects of vomiting, loss of appetite, and sleep disturbance. Attributes and levels were combined using an orthogonal design to produce a number of discrete hypothetical treatments. Parents were recruited via patient panels in different countries and asked to complete a survey. DCE data were analyzed using conditional logit models to explore the impact of each attribute on participants’ choices. Six hundred individuals (220 parents of adolescents and 380 parents of children) participated. All attributes were significant predictors of choice (*p* < 0.01). ‘Degree of symptom control’ was the most important attribute whereby the odds of choosing ‘very much improved symptoms’ compared with ‘minimally improved’ was 4.85 [95 % confidence interval (CI) = 4.28–5.49] for the adolescent group and 6.37 (95 % CI = 5.79–7.01) for the child group. Some inter-country differences emerged, e.g., achieving the best degree of symptom control was more important to parents in some countries than others. In conclusion, the study showed that duration and degree of symptom control were the most important aspects of treatment for parents in all countries. The findings revealed cultural differences in the relative importance of attributes.

**Electronic supplementary material:**

The online version of this article (doi:10.1007/s00787-013-0515-6) contains supplementary material, which is available to authorized users.

## Introduction

Stimulant treatments for attention-deficit/hyperactivity disorder (ADHD) have been shown to provide effective relief of symptoms and improve functioning and health-related quality of life (QoL) for many patients [1–3]. The therapies currently available vary in terms of chemical composition and delivery formulation, pharmacokinetic and pharmacodynamic profile, recommended dosing schedule, and efficacy profile. In addition, stimulant treatment effects can vary between individuals in relation to each of these characteristics. This may lead to different patients responding to therapies in different ways. When choosing between the different options, it is recommended that prescribing decisions take into account the preferences of parents and the affected child [2] in terms of differences in dosing schedules, efficacy profiles, and other issues.

Patient/parent preferences between different stimulant therapies may be influenced by a range of factors. In addition to the characteristics described above, different treatments for ADHD can have different side-effect rates and profiles. Common adverse events include nausea, loss of appetite, and sleep problems [2], which also vary from individual to individual. Substance abuse^1^ is common in patients diagnosed with ADHD [2], and reports have highlighted the potential for abuse of ADHD stimulant medications [4, 5]. The average age of individuals misusing ADHD stimulant medication in a US National Survey on Drug Use and Health was 19.5 years [5] and, of 1,025 students in a US university, 16 % had abused or misused medication, most commonly immediate-release methylphenidate (MPH) [4]. Students reported several reasons for misuse including improving academic achievements, sharpening attention and sustaining concentration, and reducing hyperactivity. Of 21,465 individuals who reported non-medical use of ADHD medications, 8.5 % were aged 12–17 years [5]. The potential for misuse may impact on patient/parent preferences when selecting between different therapies.

Standardized methods for eliciting an individual’s treatment preferences are widely used in healthcare research [6]. Such methods provide information regarding the importance of different attributes of interventions to determine patients’ treatment choices and levels of adherence. Preference research has examined parents’ views regarding the importance of attributes such as dosing schedules, degree of effective symptom control, modes of administration, side effects, and acceptability of treatments for ADHD [7–14]. These studies typically employed stated preference surveys [discrete choice experiments (DCEs)] to elicit parents’ preferences. Waschbusch et al. [12] conducted a DCE to explore parent preferences for the treatment of medication-naïve children. Parents were presented with descriptions of treatment combinations with varied treatment outcomes and costs. The study identified two groups of parents: the majority (70 %) preferred to avoid medication, while the remaining parents based their treatment decisions on what they perceived to be the best clinical outcomes. Further analyses predicted that parents who favored treatment in general would specifically prefer behavioral therapy, alone or in combination with medication, over stimulant medication alone. Mühlbacher et al. [8] explored six aspects of ADHD treatment with 219 participants (parents and patients) in Germany. The aim was to understand what would constitute the ideal treatment for ADHD from the point of view of parents and patients. A range of treatment attributes were studied, from improvements in QoL to variation in specific treatment characteristics (duration of effect, side effects, dosage, discretion, emotional state, and social institution). Overall, parents and patients preferred treatment that resulted in a balanced mood, being able to go out and socialize, and reduced ADHD symptoms.

Lloyd et al. [9] explored the preferences of parents of young people with ADHD in the UK regarding different MPH formulations. Parents preferred an oral, once-a-day therapy (compared with three times daily administration or a transdermal patch), rapid onset of action, and longer duration of ADHD symptom control. Glenngard et al. [13] employed DCE to assess the preferences and willingness to pay of 285 patients with ADHD (or the parents of children and adolescents) for different treatment attributes, including symptom control, side effects, dosing and cost. All attributes were significant predictors of choice (*p* < 0.01), with symptom control and side effects considered the most important treatment attributes. Conner et al. [14] explored preferences of non-stimulant ADHD medication and product profiles among children and adolescents with ADHD in the USA, and their caregivers, using a conjoint analysis survey. Treatment attributes included onset of effect, black box medication warning, improvement in oppositionality, clinician- and teacher-reported improvements in ADHD symptoms and a range of side effects. For caregivers, somnolence and improvement in oppositionality were the most important attributes. For children and adolescents themselves (aged 10–17 years), somnolence and improvement in symptoms at school were most important. Although somnolence was an important attribute, aspects relating to efficacy were generally more critical drivers of preference than non-serious side effects. These studies have demonstrated that maximizing ADHD symptom control and minimizing side effects are important to patients with ADHD and their parents [7–9, 14]. The body of DCE research to date has explored attributes that relate to functioning in an ideal ADHD treatment [8, 10], and characteristics of different MPH formulations such as speed of onset and dosing flexibility [9].

The current study extends existing knowledge in two ways. First, it includes specific treatment attributes, such as substance abuse liability and specific side effects of stimulant medication that have not been systematically explored in preference research to date. Second, it provides the first cross-national comparison of patient preferences regarding ADHD treatments, comparing responses from participants in six European countries (France, Germany, Italy, the Netherlands, Spain, and the UK) where different approaches to ADHD management may result in different ADHD treatment patterns.

## Methods

### Development of the DCE survey

A focused literature search was conducted with the specific goal of identifying important elements or attributes of ADHD stimulant medications to be included in the preference survey, including reviews of the published literature, the Food and Drug Administration (FDA)-approved package inserts/labels (US), and the Summary of Product Characteristics produced by the European Medicines Agency (EMA) for ADHD stimulant treatments. A targeted literature search was conducted in PubMed, EMBASE.com, and Google Scholar to identify articles published between 1997 and 2009 presenting evidence regarding the common side effects of treatment and information on the abuse of prescription medication in ADHD. Literature search terms included ‘treatment’ OR ‘quality of life’ OR ‘patient reported outcome’ OR ‘preference’ OR ‘clinical trial’ among other terms and ‘ADHD’ OR ‘inattention’ OR ‘hyperactivity’ OR ‘impulsivity’. The narrow scope and targeted nature of the search meant that only a limited number of highly relevant articles were retrieved. Of 12 articles initially identified, four were relevant for the present study [9, 15–17].

### Attribute identification

Potential attributes were generated from the literature review in consultation with an ADHD expert on the research team (ESB). The list was then shortened to the final study attributes based on further study team and expert review. The number of attributes was limited to seven in order to avoid an excessive number of preference item combinations, which would create an excessive burden on the participants. The attributes to be tested in the final list included: (a) duration of ADHD symptom control; (b) degree of ADHD symptom control; (c) potential for treatment abuse; (d) frequency of administration; (e) risk of vomiting; (f) risk of loss of appetite; and (g) risk of sleep disturbance.

Each attribute was described using three levels considered to reflect a relevant and realistic range for that attribute by the study team (Table 1).

**Table 1:** A list of final attributes and levels

Attribute	Description
Duration of symptom control after each dose	4–6 h
	10 h
	12 h
Degree of symptom control after each dose	Minimally improved
	Much improved
	Very much improved
Potential of treatment being abused/misused^a^	1 in 5 (20 %) adolescents
	1 in 10 (10 %) adolescents
	0 (0 %) adolescents
Frequency of medication	3 times/day
	2 times/day
	Once/day
Side effect of vomiting	1 in 10 (10 %) children/adolescents
	1 in 20 (5 %) children/adolescents
	1 in 100 (1 %) children/adolescents
Side effect of loss of appetite	1 in 10 (10 %) children/adolescents
	1 in 20 (5 %) children/adolescents
	1 in 100 (1 %) children/adolescents
Side effect of sleep disturbance	1 in 5 (20 %) children/adolescents
	1 in 10 (10 %) children/adolescents
	1 in 100 (1 %) children/adolescents

^a^This attribute was presented in the adolescent survey only because it was considered inappropriate to ask the parents of children about the risks of substance misuse

The attribute describing the potential for treatment abuse was only included in the survey for parents of adolescents (at least 13 years old). It was considered inappropriate and clinically irrelevant to ask parents of younger children about this.

### Final DCE survey

The DCE included a background questionnaire and a clinical history form. The seven attributes and three levels were combined using a published orthogonal array (available at: http://neilsloane.com/oadir/). Participants were presented with 18 pairs of hypothetical treatment choices differing systematically in terms of attributes and levels, and were asked to indicate whether they preferred treatment A or B in each choice set. Parents were also asked to categorize their child’s current treatment in terms of the attributes included in the survey, by stating which level best described their child’s current medication. All study-related documentation was translated into each target country’s local language using one forward- and one back-translation methodology in line with best practice [18]. The surveys were also culturally adapted in each country after translation. Participants in each country were debriefed and asked to comment on the accuracy of the documents. The documents were revised accordingly in each language following the cognitive debrief findings.

The survey was piloted with five parents in the UK. Parents completed the survey and were asked to comment on the clarity of the instructions, appropriateness of choices/questions, the face validity of the choices, and overall length of the survey. Following this exercise, no changes were made to the survey. The study protocol and all case-report forms including the final survey were approved by the Independent Institutional Review Board (A2106).

### Data collection

#### Sample

Parents and/or primary carers of children (aged 6–12 years) or adolescents (aged 13–17 years) with ADHD were recruited in France, Germany, Italy, the Netherlands, Spain, and the UK (*N* = 100 per country). These countries, covering Northern and Southern parts of Western Europe, were selected in order to sample broadly across differing national and cultural approaches to the diagnosis and management of ADHD. The study team aimed to recruit equally sized samples of parents of children and adolescents. The study employed a convenience sample recruited and screened by a specialist patient recruitment agency and were invited to complete the online questionnaire after giving online consent. The panel approached parents who had previously indicated that they had a child with ADHD and would be willing to take part in research. Parents reported the child’s diagnosis on his/her behalf. To minimize the possibility of recruiting non-ADHD patients in the sample, all participants completed an eligibility screener before completing the survey. This screener was designed with clinician input to help validate the sample recruitment. Further, the specialist patient recruitment agency runs continuous validation procedures to ensure the accuracy of their sample. Parents were excluded if the child or adolescent had been diagnosed with a significant psychiatric disorder (other than ADHD) that influenced the child or adolescent in a way that could not be distinguished from the impact of ADHD, including pervasive developmental disorder, Asperger’s syndrome, autism, depression, and/or conduct disorder; this determination was based on the self-report of parents. Participants received nominal compensation after completing the survey (approximately €2.50).

### Statistical analysis

Conditional logit models were estimated to explore the impact of each attribute (independent variable) on the participants’ choices (dependent variable). Analyses were separated by country and as a total sample. The conditional logit model evaluated choice responses after conditioning them on the attributes of the other treatment alternatives available within the choice set. Hence, if, for example, ‘Treatment option A’ was preferred in the choice set #1, this preference was conditional on the attributes of ‘Treatment option B’. The coefficients obtained from the logit model provided an estimate of the (log) odds ratios (ORs) of preference for treatment attributes. In addition, interactions were explored to see whether there were any country-specific differences in the preferences of parents. In the conditional logit model, participants serve as their own controls. As a result, estimates cannot be confounded by omitted between-patient covariates, making the estimates less sensitive to model specification than those based on random intercept models [19]. As there is no heterogeneity within an individual on time invariant covariates, the baseline characteristics are absorbed within individual-level fixed effects. All analyses were conducted in Stata vs 9.0.

## Results

### Participant characteristics

A total of 600 participants (220 parents of adolescents and 380 parents of children with ADHD) completed all study questionnaires. Demographics and background clinical data are presented by country (Table 2). Overall, the majority of respondents in both cohorts were female (61.8 and 59.7 %), with a modal age ranging from 30 to 44 years. The age ranges of adolescents and children were similar in all countries. Parents reported a wide range of different treatments in use by their children/adolescents. The most commonly used treatments were long- and short-acting MPH, particularly in the UK (adolescent 85 %, child 94 %), the Netherlands (adolescent 89 %, child 94 %), and Germany (adolescent 88 %, child 99 %). Amphetamine use was higher in Spain, Italy, and France compared with the UK, Germany, and Netherlands (Table 2). More than half of all patients had also received non-pharmacological therapy. The majority of parents of adolescents (66 %) reported that they were aware that some ADHD medications have abuse potential.

**Table 2:** Socio-demographic profile of the sample by country (short version)

	Country												Total	
	UK		Netherlands		Germany		Spain		Italy		France			
	Adol (*N* = 52)	Child (*N* = 48)	Adol (*N* = 36)	Child (*N* = 64)	Adol (*N* = 33)	Child (*N* = 67)	Adol (*N* = 38)	Child (*N* = 62)	Adol (*N* = 31)	Child (*N* = 69)	Adol (*N* = 30)	Child (*N* = 70)	Adol (*N* = 220)	Child (*N* = 380)
Female parent	35 (67.3)	29 (60.4)	27 (75.0)	48 (75.0)	25 (75.8)	42 (62.7)	23 (60.5)	30 (48.4)	9 (29.0)	36 (52.2)	17 (56.7)	42 (60.0)	136 (61.8)	227 (59.7)
Age range of parent, years	40–44	35–39	40–44	35–39	40–44	35–39	35–39	30–34	35–39	35–39	40–44	35–39	35–44	30–39
Employment status														
Working full time	23 (44.2)	21 (43.8)	11 (30.6)	14 (21.9)	11 (33.3)	34 (50.7)	20 (52.6)	40 (64.5)	24 (77.4)	50 (72.5)	25 (83.3)	52 (74.3)	114 (51.8)	211 (55.5)
Working part time	8 (15.4)	11 (22.9)	16 (44.4)	27 (42.2)	13 (39.4)	14 (20.9)	9 (23.7)	11 (17.7)	5 (16.1)	7 (10.1)	4 (13.3)	10 (14.3)	55 (25.0)	80 (21.1)
Student	0	1 (2.1)	1 (2.8)	0	0	0	3 (7.9)	1 (1.6)	0	1 (1.4)	0	3 (4.3)	4 (1.8)	6 (1.6)
Home-maker	19 (36.5)	11 (22.9)	2 (5.6)	19 (29.7)	5 (15.2)	16 (23.9)	2 (5.3)	4 (6.5)	2 (6.5)	8 (11.6)	0	4 (5.7)	30 (13.6)	62 (16.3)
Unemployed	1 (1.9)	3 (6.3)	5 (13.9)	3 (4.7)	4 (12.1)	3 (4.5)	4 (10.5)	6 (9.7)	0	1 (1.4)	1 (3.3)	1 (1.4)	15 (6.8)	17 (4.5)
Prefer not to answer	1 (1.9)	1 (2.1)	1 (2.8)	1 (1.6)	0	0	0	0	0	2 (2.9)	0	0	2 (0.9)	4 (1.1)
Education														
16 year examinations	17 (32.7)	19 (39.6)	10 (27.8)	32 (50.0)	4 (12.1)	5 (7.5)	1 (2.6)	4 (6.5)	0	7 (10.1)	1 (3.3)	1 (1.4)	33 (15.0)	68 (17.9)
Vocational qualification	6 (11.5)	5 (10.4)	14 (38.9)	22 (34.4)	16 (48.5)	35 (52.2)	3 (7.9)	1 (1.6)	5 (16.1)	7 (10.1)	1 (3.3)	6 (8.6)	43 (19.5)	76 (20.0)
Completed high school	10 (19.2)	7 (14.6)	3 (8.3)	4 (6.3)	9 (27.2)	12 (17.9)	13 (34.2)	19 (30.6)	6 (19.4)	17 (24.6)	9 (30.0)	15 (21.4)	44 (20.0)	74 (19.5)
Under-graduate degree	8 (15.4)	8 (16.7)	6 (16.7)	3 (4.7)	1 (3.0)	3 (4.5)	15 (39.5)	23 (37.1)	5 (16.1)	11 (15.9)	7 (23.3)	27 (38.6)	42 (19.1)	75 (19.7)
Post-graduate degree	6 (11.5)	9 (18.8)	3 (8.3)	1 (1.6)	3 (9.1)	11 (16.4)	6 (15.8)	9 (14.5)	15 (48.4)	23 (33.3)	12 (40.0)	21 (30.0)	45 (20.5)	74 (19.5)
Other	0	0	0	1 (1.6)	0	1 (1.5)	0	6 (9.7)	0	4 (5.8)	0	0	0	12 (3.2)
No formal qualification	5 (9.6)	0	0	1 (1.6)	0	0	0	0	0	0	0	0	5 (2.3)	1 (0.3)
Child’s age, mean (SD)	14.4 (1.3)	9.6 (1.9)	14.9 (1.4)	9.9 (1.6)	14.4 (1.0)	9.6 (1.9)	14.4 (1.1)	9.2 (1.9)	14.5 (1.1)	9.1 (2.2)	14.6 (1.2)	9.3 (2.1)	14.5 (1.2)	9.5 (2.0)
Age at diagnosis, years, mean (SD)	6.6 (3.9)	5.3 (3.2)	8.1 (3.2)	5.9 (2.9)	5.6 (3.8)	5.7 (2.2)	6.3 (4.2)	4.3 (3.9)	7.7 (4.8)	3.0 (4.8)	7.1 (4.1)	4.1 (3.7)	6.9 (4.1)	4.7 (3.7)
Time from first symptoms to diagnosis, years, mean (SD)	3.2 (3.7)	1.9 (2.6)	2.9 (2.7)	2.5 (2.4)	2.0 (2.8)	1.2 (1.4)	0.9 (1.5)	0.8 (1.3)	0.4 (0.7)	0.7 (2.2)	0.9 (2.3)	1.0 (1.8)	1.9 (2.8)	1.3 (2.1)
Time from diagnosis to medication, months, mean (SD)	9.6 (19.0)	4.3 (8.9)	7.3 (16.3)	4.4 (7.2)	11.5 (18.2)	9.9 (14.6)	3.7 (7.2)	4.6 (15.6)	2.7 (4.2)	5.5 (11.4)^a^	3.0 (5.4)	3.2 (5.7)	6.6 (14.2)	5.4 (11.3)^a^
Child receives additional therapy currently^b^	30 (57.7)	29 (60.4)	19 (52.8)	37 (57.8)	20 (60.6)	56 (83.6)	24 (63.2)	50 (80.6)	23 (74.2)	49 (71.0)	22 (73.3)	56 (80.0)	138 (62.7)	277 (72.9)
Current medication														
Methylphenidate	44 (84.6)	45 (93.8)	32 (88.9)	60 (93.8)	29 (87.9)	66 (98.5)	22 (57.9)	42 (67.7)	27 (87.1)	41 (59.4)	17 (56.7)	44 (62.9)	171 (77.7)	298 (78.4)
Amphetamine	8 (15.4)	5 (10.4)	1 (2.8)	4 (6.3)	0	0	19 (50)	20 (32)	15 (48)	42 (61)	10 (33)	22 (29)	50 (23)	90 (24)
Non-stimulants	9 (17)	10 (21)	2 (6)	5 (8)	11 (33)	15 (22)	13 (34)	15 (24)	21 (68)	41 (59)	8 (27)	25 (36)	64 (29)	120 (32)
Other medication	3 (5.8)	3 (6.3)	4 (11.1)	6 (9.4)	0	0	1 (2.6)	3 (4.8)	1 (3.2)	3 (4.3)	0	1 (1.4)	9 (4.1)	16 (4.2)
Previous ADHD medication	24 (46.2)	5 (10.4)	17 (47.2)	18 (28.1)	23 (69.7)	22 (32.8)	10 (26.3)	14 (22.6)	5 (16.1)	15 (21.7)	2 (6.7)	13 (18.6)	81 (36.8)	87 (22.9)
Aware of abuse potential?	35 (67.3)	N/A	29 (80.6)	N/A	25 (75.8)	N/A	25 (65.8)	N/A	15 (48.4)	N/A	17 (56.7)	N/A	146 (66.4)	N/A

Data are *N* (%) unless indicated otherwise

*N/A* not applicable

^a^Missing data

^b^Additional therapy refers to all other therapy excluding medication

Parents’ perception and ratings of ADHD current treatment attributes are presented in Table 3. Approximately 40–45 % of parents reported that the treatment effect lasted for 4–6 h each day. At least 39 % of parents reported that patients were taking ADHD medication once daily; however, only 22 % of parents of adolescents and 16 % of parents of children reported that the effect of ADHD medication taken by their child lasted for 12 h. The least common dosing regimen was three times daily across the whole sample, and more adolescents were prescribed a once-daily formulation compared with children. In Spain, France, and Italy, 19–35 % of parents reported that their child or adolescent experienced vomiting due to their ADHD medication. High rates of sleep disturbance (total adolescents 41 % and children 45 %) and appetite loss (total adolescents 46 % and children 43 %) were also reported.

**Table 3:** Current treatment and side effect profile of medication by country

	Country													
	UK		Netherlands		Germany		Spain		Italy		France		Total	
	Adol (*N* = 52)	Child (*N* = 48)	Adol (*N* = 36)	Child (*N* = 64)	Adol (*N* = 33)	Child (*N* = 67)	Adol (*N* = 38)	Child (*N* = 62)	Adol (*N* = 31)	Child (*N* = 69)	Adol (*N* = 30)	Child (*N* = 70)	Adol (*N* = 220)	Child (*N* = 380)
How long effects last, hours														
4–6 h	25 (48.1)	22 (45.8)	12 (33.3)	34 (53.1)	11 (33.3)	32 (47.8)	12 (31.6)	20 (32.3)	14 (45.2)	31 (44.9)	12 (40.0)	31 (44.3)	86 (39.1)	170 (44.7)
10 h	16 (30.8)	19 (39.6)	12 (33.3)	15 (23.4)	17 (51.5)	25 (37.3)	17 (44.7)	28 (45.2)	12 (38.7)	29 (42.0)	12 (40.0)	32 (45.7)	86 (39.1)	148 (38.9)
12 h	11 (21.2)	7 (14.6)	12 (33.3)	14 (21.9)	5 (15.2)	10 (14.9)	9 (23.7)	13 (21.0)	5 (16.1)	9 (13.0)	6 (20.0)	7 (10.0)	48 (21.8)	60 (15.8)
Frequency (per day)														
3 times	12 (23.1)	11 (22.9)	7 (19.4)	25 (39.1)	0	7 (10.4)	5 (13.2)	11 (17.7)	2 (6.5)	7 (10.1)	2 (6.7)	14 (20.0)	28 (12.7)	75 (19.7)
2 times	9 (17.3)	18 (37.5)	9 (25.0)	13 (20.3)	4 (12.1)	18 (26.9)	19 (50.0)	32 (51.6)	16 (51.6)	41 (59.4)	18 (60.0)	34 (48.6)	75 (34.1)	156 (41.1)
Once	31 (59.6)	19 (39.6)	20 (55.6)	26 (40.6)	29 (87.9)	42 (62.7)	14 (36.8)	19 (30.6)	13 (41.9)	21 (30.4)	10 (33.3)	22 (31.4)	117 (53.2)	149 (39.2)
Side effect														
Vomiting	3 (5.8)	8 (16.7)	1 (2.8)	4 (6.3)	1 (3.0)	5 (7.5)	8 (21.1)	18 (29.0)	6 (19.4)	24 (34.8)	9 (30.0)	21 (30.0)	28 (12.7)	80 (21.1)
Loss of appetite	29 (55.8)	21 (43.8)	11 (30.6)	30 (46.9)	22 (66.7)	32 (47.8)	18 (47.4)	26 (41.9)	12 (38.7)	23 (33.3)	9 (30.0)	33 (47.1)	101 (45.9)	165 (43.4)
Sleep disturbance	22 (42.3)	26 (54.2)	11 (30.6)	30 (46.9)	10 (30.3)	20 (29.9)	15 (39.5)	32 (51.6)	18 (58.1)	25 (36.2)	14 (46.7)	36 (51.4)	90 (40.9)	169 (44.5)

Data are given as *N* (%)

### DCE results

Table 4 presents the results of the DCE for parents’ treatment choices in terms of ORs and associated 95 % confidence intervals (95 % CIs), showing the order of importance of a given attribute when selecting a treatment. The analyses indicated the preference for different attributes and improvements in the levels of those attributes for ADHD therapies. The findings had face validity in that parents preferred treatments that improved outcomes or reduced side effects or risks, and all attributes were significant predictors of choice.

**Table 4:** Results of the logit model by adolescent and child groups, in order of importance of attributes (most important to least important)

Attribute^a^	Adolescent		Child	
	OR (SE)^b^	95 % CI	OR (SE)^b^	95 % CI
Degree of symptom control				
Reference group: minimally improved				
Much improved	2.70 (0.16)	2.41, 3.03	3.61 (0.17)	3.3, 3.95
Very much improved	4.85 (0.31)	4.28, 5.49	6.37 (0.31)	5.79, 7.01
Duration of symptom control				
Reference group: 4–6 h				
10 h	1.38 (0.08)	1.22, 1.55	1.34 (0.06)	1.23, 1.47
12 h	1.59 (0.10)	1.41, 1.79	1.60 (0.08)	1.46, 1.76
Loss of appetite				
1 % increase in risk of loss of appetite	0.98 (0.01)	0.97, 0.99	0.95 (0.01)	0.94, 0.96
Potential of treatment abuse				
1 % increase in potential of treatment abuse	0.97 (0.00)	0.97, 0.98	–	–
Vomiting				
1 % increase in risk of vomiting	0.97 (0.01)	0.96, 0.98	0.96 (0.01)	0.95, 0.97
Sleep disturbance				
1 % increase in risk of sleep disturbance	0.96 (0.00)	0.95, 0.97	0.96 (0.02)	0.95, 0.96
Frequency of medication				
One additional administration in dosage per day	0.89 (0.03)	0.83, 0.94	0.86 (0.02)	0.82, 0.90

*CI* confidence interval, *OR* odds ratio, *SE* standard error

^a^In this model, the attributes are the independent variables and parents’ choice data are the dependent variable. All attributes are significant predicators (*p* < 0.01)

^b^Standard error shows whether the ORs are significantly different from 1

‘Degree of symptom control’ of ADHD medication was the attribute most highly valued by parents in both groups. Parents of adolescents were nearly five times more likely to choose medications that demonstrated ‘very much improved’ symptoms compared with medications that showed only ‘minimally improved’ symptoms (reference level) (OR = 4.85, 95 % CI = 4.28–5.49). For the child group, this preference was even stronger (OR = 6.37, 95 % CI = 5.79–7.01).

A comparison of the child and adolescent groups showed that the attribute ‘duration of symptom control’ of ADHD was approximately equally important for both sets of parents. Parents preferred 12 h of symptom control to therapies with a shorter duration of action. Compared with a duration of symptom control of 4–6 h, both sets of parents were 1.6 times more likely to prefer a medication with a duration of symptom control of 12 h (*p* < 0.01). The impact of each of the three side effects on treatment preferences was similar for the two parent groups. A 1 % increase in the probability of any side effect reduced the likelihood of choosing a treatment by 30–50 %. Loss of appetite was considered the most important side effect to avoid (adolescent OR = 0.98, 95 % CI = 0.97–0.99; child OR = 0.95, 95 % CI = 0.94–0.96) and sleep disturbance was considered the least important (adolescent OR = 0.96, 95 % CI = 0.95–0.97; child OR = 0.96, 95 % CI = 0.95–0.96) by parents of both groups. Parents of adolescents reported a significant preference for a treatment with a reduced risk of substance abuse (OR = 0.97, 95 % CI = 0.97–0.98). Parents were 30 % less likely to choose a treatment associated with a 10 % increase in the risk of substance abuse. ‘Frequency of medication’ had a significant but relatively smaller impact on parents’ treatment choices. Treatments that required an additional administration per day reduced the likelihood that parents would choose that treatment by 11 % for adolescents (OR = 0.89, 95 % CI = 0.83–0.94) and by 14 % for children (OR = 0.86, 95 % CI = 0.82–0.90).

### Inter-country differences

Table 5 shows the ORs of the significant attributes for parents of adolescents and children broken down by country. Data in this table demonstrate that parents in each country had different patterns of preferences compared with UK parents, who were used as a reference case for analytical purposes. Parents in different countries assigned different levels of importance to different aspects of treatments.

**Table 5:** Results of the logit model demonstrating country differences by adolescent and child groups in order of preference (only significant results are shown)

	Adolescents		Children	
	OR (SE)	95 % CI	OR (SE)	95 % CI
Country^a^ (reference: UK^b^)				
Netherlands	2.92 (1.19)	1.31, 6.48	–	–
Germany	9.87 (5.01)	3.65, 26.7	–	–
Spain	–	–	0.55 (0.17)	0.31, 1.00
Degree of symptom control (reference: UK)				
Much improved	3.68 (0.47)	2.88, 4.72	3.75 (0.49)	2.91, 4.84
Very much improved	8.45 (1.18)	6.44, 11.11	5.87 (0.81)	4.48, 7.68
Spain × much improved symptoms	0.64 (0.12)	0.44, 0.92		
Spain × very much improved symptoms	0.39 (0.08)	0.26, 0.58	1.55 (0.29)	1.07, 2.23
Italy × much improved symptoms	0.45 (0.09)	0.31, 0.66	0.56 (0.09)	0.41, 0.77
Italy × very much improved symptoms	0.27 (0.06)	0.18, 0.4	0.50 (0.09)	0.36, 0.71
France × much improved symptoms	0.54 (0.11)	0.37, 0.8	–	–
France × very much improved symptoms	0.38 (0.08)	0.25, 0.58	–	–
Duration of symptom control (reference: UK)				
10 h	1.49 (0.19)	1.16, 1.91	1.52 (0.20)	1.17, 1.95
12 h	1.88 (0.25)	1.45, 2.44	1.81 (0.24)	1.39, 2.34
Italy × 12 h symptom control	–	–	0.66 (0.11)	0.48, 0.92
Potential of treatment abuse (reference: UK)				
UK 1 % increase in potential of treatment abuse	0.97 (0.01)	0.96, 0.99	N/A	N/A
Vomiting (reference: UK)				
1 % increase in risk of vomiting	0.97 (0.01)	0.94, 0.99	–	–
Netherlands × 1 % increase in risk	0.95 (0.02)	0.91, 1	0.96 (0.02)	0.92, 0.99
Germany × 1 % increase in risk	0.92 (0.03)	0.87, 0.98	–	–
Sleep disturbance (reference: UK)				
1 % increase in risk of sleep disturbance	0.96 (0.01)	0.95, 0.97	–	–
Netherlands × 1 % increase in risk	0.97 (0.01)	0.95, 0.99	–	–
France × 1 % increase in risk	1.03 (0.01)	1.01, 1.05	1.52 (0.20)	1.17, 1.95
Germany × 1 % increase in risk	–	–	0.55 (0.17)	0.31, 1.00
Loss of appetite (reference: UK)				
1 % increase in risk of loss of appetite			0.96 (0.01)	0.95, 0.97
Netherlands × 1 % increase in risk	0.93 (0.02)	0.88, 0.97	–	–
Germany × 1 % increase in risk	0.91 (0.03)	0.85, 0.97	0.97 (0.01)	0.95, 0.99
Frequency of medication (reference: UK)				
One additional administration in dosage per day	0.79 (0.05)	0.69, 0.9	0.95 (0.01)	0.93, 0.98
Germany × one additional administration in dosage	0.60 (0.08)	0.46, 0.79	–	–
Spain × one additional administration in dosage	1.37 (0.13)	1.13, 1.66	–	–
Italy × one additional administration in dosage	1.39 (0.14)	1.14, 1.69	1.06 (0.02)	1.02, 1.10
France × one additional administration in dosage	1.25 (0.13)	1.02, 1.53	–	–
Netherlands × one additional administration in dosage			0.95 (0.02)	0.91, 0.99

*CI* confidence interval, *OR* odds ratio, *SE* standard error

This table shows the ORs of the significant attributes by country only in each group. In this table, the country is the dependent variable. Only countries that were significantly different from the UK (reference case) and the attributes that were most important to parents in each country are shown. For example, Spanish parents were more concerned than UK parents about the need to take medication more than once per day (OR = 1.37, 95 % CI = 1.13–1.66)

^a^Countries with a significant difference in overall patient preference compared with the UK, irrespective of attributes

^b^UK is base case profile

#### Adolescent sample

Spanish, Italian, and French parents all placed less value on the degree of symptom control (Spain, OR = 0.39, 95 % CI = 0.26–0.58; Italy, OR = 0.27, 95 % CI = 0.18–0.40; France, OR = 0.38, 95 % CI = 0.25–0.58) across all levels compared with parents in Germany, the Netherlands, and the UK. ‘Duration of symptom control’ and ‘potential of substance abuse’ were equally important across all countries for parents of adolescents. Dutch and German parents were also more concerned than other parents in general about the experience of side effects such as vomiting (Netherlands, OR = 0.95, 95 % CI = 0.91–1.00; Germany, OR = 0.92, 95 % CI = 0.87–0.98) and loss of appetite (Netherlands, OR = 0.93, 95 % CI = 0.88–0.97; Germany, OR = 0.91, 95 % CI = 0.85–0.97). Dutch and French parents of adolescents were more concerned than UK parents about sleep disturbance associated with medication (Netherlands, OR = 0.97, 95 % CI = 0.95–0.99; France, OR = 1.03, 95 % CI = 1.01–1.05). For the ‘frequency of medication’ attribute, German parents stated a stronger preference (compared with UK parents) to avoid a treatment that needed to be taken more than once daily (OR = 0.60, 95 % CI = 0.46–0.79). In contrast, Spanish (OR = 1.37, 95 % CI = 1.13–1.66), Italian (OR = 1.39, 95 % CI = 1.14–1.69), and French (OR = 1.25, 95 % CI = 1.02–1.53) parents were less concerned than British parents about the number of times per day that their child required medication.

#### Child sample

Parents of children in Italy placed less value on the duration and degree of symptom control than British parents (this was consistent with the findings from the adolescent parents). Achieving the best level of symptom control was important for parents of children in Spain and the UK, with British parents placing most value on this attribute. Dutch and German parents of both groups were most concerned with side effects of medication (vomiting, Netherlands: OR = 0.96, 95 % CI = 0.92–0.99; loss of appetite, Germany: OR = 0.97, 95 % CI = 0.95–0.99; sleep disturbance, Germany: OR = 0.55, 95 % CI = 0.31–1.00). Dutch parents placed similar importance on the need to take more than one tablet per day as British parents (OR = 0.95, 95 % CI = 0.91–0.99). When comparing the two groups in Italy, parents of children were less concerned about the need to take more than one tablet per day than parents of adolescents (child, OR = 1.06, 95 % CI = 1.02–1.10; adolescent, OR = 1.39, 95 % CI = 1.14–1.69). Spanish parents of children placed more value on the best level of symptom control, while the Germans were more concerned about loss of appetite as a side effect.

## Discussion

This study is the first large-scale pan-European, cross-national comparison of parental preferences for all stimulant treatments for ADHD licensed within Europe. Our analysis considered a broader range of medication attributes than has been explored in previous studies, including the degree and duration of ADHD symptom control, treatment side effects, and perceived risks and concerns of abuse potential in shaping parental preference for selecting one ADHD medication versus another. Information on the participants’ current medication was also collected, which allowed comparisons between the hypothetical treatment preferences and the current medication profile. The parent preference data were also examined at a country level. These analyses often indicated that preferences of parents in the Northern European countries (UK, Germany, and the Netherlands) were similar and these preferences differed from those of parents in the Southern European countries (Italy, Spain, and France).

Parents rated symptom control as the most important treatment attribute, with both degree of control and its duration influential in determining preferences. These findings are consistent with previous work by our group and by Mühlbacher et al. [8], which indicated that a long duration of symptom control is important in determining parental preferences when they are selecting treatment for ADHD. In the present study, the degree of control was more important than its duration. A relatively similar pattern was seen across all countries; however, in absolute terms, there were some differences between Northern and Southern European countries. In adolescents, parents from the Northern European countries placed more importance on the degree of symptom control. This preference was not reflected by the degree of symptom control currently experienced in real life by many patients. Although more than 40 % of patients were taking once-daily ADHD medications, <25 % of parents reported that the effect of ADHD medication that their child was taking during the study lasted for 12 h. The fact that parents were more likely to prefer a medication with a longer duration of symptom control suggests that there is a degree of unmet need in relation to this particular treatment goal.

There were some cross-national differences in relation to this second aspect of symptom control and the need/preference for multiple dosing of short-acting medications. Respondents in Southern European countries were less concerned about the need to take more than one tablet per day. It is possible that this reflects cross-European lifestyle differences. Where families take a longer break during the middle of the day for lunch, it may be more feasible to administer a second dose of treatment. Parents in Southern European countries, particularly outside of the big cities, may be more likely to take a longer break during the day than parents in the Northern European countries. A question for manufacturers, therefore, arises as to how new formulations and delivery mechanisms can extend the duration of symptom control without leading to an unacceptable increase in side effects like sleep disturbances. A similar question is also relevant to clinicians, in terms of how they can combine existing therapies to extend the duration of symptom control of the medication. Treatment choices are always based on a balance between the positive attributes of treatments and the negative aspects.

Other attributes of treatment were significant drivers of parent choice. The current DCE method allowed evaluation of the extent to which parents are willing to trade the benefits of treatments against their risks. In general, despite symptom control being the most important determining factor when it comes to treatment preferences, parents were willing to accept some reduction in symptom control in order to avoid even a relatively small increase in side effects or risk of substance abuse. Both symptom control and side effects were independently significant predictors of choice; therefore, it is possible to estimate the marginal rates of substitution between these attributes. The data also demonstrate that parents would prefer a treatment with equal efficacy but no associated risk of abuse or side effects. Previous studies have reported similar findings, whereby efficacy was regarded as the most important attribute; however, side effects and dosing were also identified as key aspects of treatment [13, 14].‘Potential of treatment abuse’ was considered an important attribute by parents of adolescents. Parents gave this attribute more importance than avoidance of side effects such as sleep disturbance and vomiting when choosing a treatment. No cross-country differences emerged in the value that parents placed on reducing the risk of abuse potential. Other data from the survey (Supplementary Table 1) indicated that parents in Southern European countries were more likely to be concerned about the potential for abuse related to treatment than parents in other countries. Higher levels of awareness and concern would be expected to translate into a stronger preference to avoid such treatments among parents in Southern European countries. The data indicate that this was an important concern for all parents and they all placed value on a treatment that avoided the risk of abuse potential. Overall, Dutch and German parents were more concerned than parents from other countries about side effects and had a stronger preference to avoid them. This was true for both vomiting and loss of appetite. Parents in France, the Netherlands, and Germany also placed more value on avoiding treatments that could cause sleep disturbance.

Some other interesting differences emerged between the parents in the different countries in terms of their experience of ADHD and the care their children had received. There was evidence of a shorter length of time between the first appearance of symptoms to the diagnosis of ADHD in Southern European countries. At least in this dataset diagnoses were made at a younger age in these countries. Given the sampling approach taken, it is difficult to determine whether this difference is a real cultural effect or rather something specific to the particular samples recruited for this study. The analyses also identified some differences in prescribing patterns. The Northern European countries reported higher use of MPH, whereas the Southern European countries reported a more varied range of treatments, particularly amphetamine-based and non-stimulant products. This may reflect different patterns of medication availability and treatment patterns in the different European countries [20].

There are some important limitations to this study that should be considered when interpreting the results. The sample was obtained through a specialist patient recruitment agency and, therefore, it was a self-selected group that may not have been completely representative of the European population. This could bias the findings and affect the results in different ways in different countries. In addition, parents/caregivers who participated in the study may have children/adolescents with more severe symptoms, which may partially explain why participants reported a higher use of baseline amphetamine in the sample than in the general ADHD population. The mean age at diagnosis was lower than expected in this study. This could partially be explained by the fact that diagnosis of ADHD was gathered from parents and not confirmed with patient medical records. Thus, the low age of diagnosis may be confounded by parental recall bias that cannot be confirmed with patient medical records.

The fact that the survey was performed online may have precluded some people from taking part, i.e., participants with no internet access or who do not know how to use the internet. This may have biased the type of respondents, e.g., towards a younger or more educated population, although all efforts were made to recruit a sample representative of the population in terms of age and gender in each country. Practical constraints required the research team to limit the number of attributes that were included in the DCE in order to avoid an overly complex survey. Consequently, the choices that parents were asked to make may be a simplification of actual, real-life treatment choices. However, the survey was not designed to capture information on all aspects of ADHD treatment; rather, an assessment of key factors/attributes that is more meaningful to parents when they are faced with decisions to choose the most appropriate therapy among the stimulant class was wanted. In fact, this survey focused on the attributes of stimulant medications only and, therefore, there is the possibility that only benefits unique to this specific class of medications were captured. The attribute describing duration was expressed in terms of the number of hours per day of symptom control. The attribute did not specify at what time in the day the treatment would provide symptom control (e.g. morning or afternoon). This may be an important issue to examine in future studies.

The findings of this study may provide insight to clinicians, regulatory bodies and decision makers regarding the key attributes that drive patient/parent preferences for the choice of ADHD treatments across countries. Importantly, it is acknowledged that poor patient/parent satisfaction with pharmacotherapy may lead to poor adherence to treatment, which could have a negative impact on clinical outcomes [2]. Further, our results indicate what preferences and attributes should be considered for the development of new ADHD medications. Indeed, the disconnection between current treatment options and stated parental preferences suggests the existence of unmet needs in the treatment of ADHD.

## Conclusions

This study identified parent preferences for different aspects of ADHD treatment in six European countries. Duration and degree of symptom control were the most important aspects of treatment for parents in all countries; however, the findings revealed cultural differences in the relative importance of other attributes. The DCE data also reveal participants’ preferences for attributes in relation to administration, potential of treatment abuse, and tolerance of side effects. The DCE method allows clinicians and decision makers to identify the key aspects of treatment that are important to parents, which may lead to better tailoring of treatments and improved adherence, persistence, and patient/caregiver relevant outcomes.

## Electronic supplementary material

Below is the link to the electronic supplementary material.

Supplementary material 1 (DOCX 70 kb)

## Footnotes

1 Substance abuse is defined as ‘a person regularly taking a drug (in this case a prescription treatment) to alter their mood or state of consciousness’ (www.nhs.uk/conditions/drug-misuse/).
