# Supplementary material for: Parent preferences regarding stimulant therapies for ADHD: a comparison across six European countries
Source: Eur Child Adolesc Psychiatry. 2014 Jan 18;23(12):1189–200. doi: 10.1007/s00787-013-0515-6 (PMC4246123; doi:10.1007/s00787-013-0515-6)
Supplement: Supplementary file 1 — Supplementary material 1 (DOCX 70 kb) [file 787_2013_515_MOESM1_ESM.docx]

**Supplementary Table I. Socio-demographic profile of the sample in each country (long version)**

|  | Country | | | | | | | | | | | | | Total | |
| --- | --- | --- | --- | --- | --- | --- | --- | --- | --- | --- | --- | --- | --- | --- | --- |
|  | UK | | Netherlands | | Germany | | Spain | | Italy | | France | | |  |  |
|  | Adol | Child | Adol | Child | Adol | Child | Adol | Child | Adol | Child | Adol | Child | Adol | | Child |
|  | (N=52) | (N=48) | (N=36) | (N=64) | (N=33) | (N=67) | (N=38) | (N=62) | (N=31) | (N=69) | (N=30) | (N=70) | (N=220) | | (N=380) |
| Parent has more than 1 child with ADHD | 3  (5.8) | 2  (4.2) | 8  (22.2) | 4  (6.3) | 4  (12.1) | 9  (13.4) | 1  (2.6) | 1  (1.6) | 4  (12.9) | 10 (14.4) | 1  (3.3) | 5  (7.1) | 21  (9.5) | | 31  (8.1) |
| Female parent | 35  (67.3) | 29 (60.4) | 27 (75.0) | 48 (75.0) | 25 (75.8) | 42 (62.7) | 23 (60.5) | 30 (48.4) | 9  (29.0) | 36 (52.2) | 17 (56.7) | 42 (60.0) | 136 (61.8) | | 227 (59.7) |
| Age range of parent | 40–44 | 35–39 | 40–44 | 35–39 | 40–44 | 35–39 | 35–39 | 30–34 | 35–39 | 35–39 | 40–44 | 35–39 | 35–39 | | 35–39 |
| Marital status |  |  |  |  |  |  |  |  |  |  |  |  |  | |  |
| Single | 5  (9.6) | 9  (18.8) | 2  (5.6) | 2  (3.1) | 2  (6.1) | 12 (17.9) | 4  (10.5) | 13 (21.0) | 5  (16.1) | 15 (21.7) | 2  (6.7) | 3  (4.3) | 20  (9.1) | | 54  (14.2) |
| Married/living with partner | 42  (80.8) | 37 (77.1) | 26 (72.2) | 52 (81.3) | 21 (63.6) | 49 (73.1) | 28 (73.7) | 42 (67.7) | 23 (74.2) | 47 (68.1) | 23 (76.7) | 61 (87.1) | 163 (74.1) | | 288 (75.8) |
| Separated/ divorced | 5  (9.6) | 2  (4.2) | 7  (19.4) | 10 (15.6) | 10 (30.3) | 6  (9.0) | 4  (10.5) | 7  (11.3) | 3  (9.7) | 4  (5.8) | 5 (16.7) | 5  (7.1) | 34 (15.5) | | 34  (8.9) |
| Prefer not to answer | 0 | 0 | 1  (2.8) | 0 | 0 | 0 | 2  (5.3) | 0 | 0 | 3  (4.3) | 0 | 1  (1.4) | 3  (1.4) | | 4  (1.1) |
| Employment status | |  |  |  |  |  |  |  |  |  |  |  |  | |  |
| Working full time | 23  (44.2) | 21 (43.8) | 11  (30.6) | 14 (21.9) | 11 (33.3) | 34 (50.7) | 20  (52.6) | 40 (64.5) | 24 (77.4) | 50 (72.5) | 25 (83.3) | 52 (74.3) | 114 (51.8) | | 211 (55.5) |
| Working part time | 8  (15.4) | 11 (22.9) | 16  (44.4) | 27 (42.2) | 13  (39.4) | 14 (20.9) | 9  (23.7) | 11 (17.7) | 5  (16.1) | 7  (10.1) | 4 (13.3) | 10 (14.3) | 55 (25.0) | | 80 (21.1) |
| Student | 0 | 1 (2.1) | 1 (2.8) | 0 | 0 | 0 | 3 (7.9) | 1 (1.6) | 0 | 1 (1.4) | 0 | 3 (4.3) | 4 (1.8) | | 6 (1.6) |
| Home-maker | 19  (36.5) | 11 (22.9) | 2  (5.6) | 19 (29.7) | 5  (15.2) | 16 (23.9) | 2  (5.3) | 4  (6.5) | 2  (6.5) | 8  (11.6) | 0 | 4  (5.7) | 30 (13.6) | | 62  (16.3) |
| Unemployed | 1 (1.9) | 3 (6.3) | 5 (13.9) | 3 (4.7) | 4 (12.1) | 3 (4.5) | 4 (10.5) | 6 (9.7) | 0 | 1 (1.4) | 1 (3.3) | 1 (1.4) | 15 (6.8) | | 17 (4.5) |
| Prefer not to answer | 1 (1.9) | 1 (2.1) | 1 (2.8) | 1 (1.6) | 0 | 0 | 0 | 0 | 0 | 2 (2.9) | 0 | 0 | 2 (0.9) | | 4 (1.1) |
| Education |  |  |  |  |  |  |  |  |  |  |  |  |  | |  |
| 16 yr exams | 17  (32.7) | 19 (39.6) | 10 (27.8) | 32 (50.0) | 4  (12.1) | 5  (7.5) | 1  (2.6) | 4  (6.5) | 0 | 7  (10.1) | 1  (3.3) | 1  (1.4) | 33 (15.0) | | 68  (17.9) |
| Vocational qualification | 6  (11.5) | 5  (10.4) | 14 (38.9) | 22 (34.4) | 16 (48.5) | 35 (52.2) | 3  (7.9) | 1  (1.6) | 5  (16.1) | 7  (10.1) | 1  (3.3) | 6  (8.6) | 43 (19.5) | | 76  (20.0) |
| Completed high school | 10  (19.2) | 7  (14.6) | 3  (8.3) | 4  (6.3) | 9  (27.2) | 12 (17.9) | 13 (34.2) | 19 (30.6) | 6  (19.4) | 17 (24.6) | 9 (30.0) | 15 (21.4) | 44 (20.0) | | 74  (19.5) |
| Under-graduate degree | 8  (15.4) | 8  (16.7) | 6  (16.7) | 3  (4.7) | 1  (3.0) | 3  (4.5) | 15 (39.5) | 23 (37.1) | 5  (16.1) | 11 (15.9) | 7 (23.3) | 27 (38.6) | 42 (19.1) | | 75  (19.7) |
| Post-graduate degree | 6  (11.5) | 9  (18.8) | 3  (8.3) | 1  (1.6) | 3  (9.1) | 11 (16.4) | 6  (15.8) | 9  (14.5) | 15 (48.4) | 23 (33.3) | 12 (40.0) | 21 (30.0) | 45 (20.5) | | 74  (19.5) |
| Other | 0 | 0 | 0 | 1 (1.6) | 0 | 1 (1.5) | 0 | 6 (9.7) | 0 | 4 (5.8) | 0 | 0 | 0 | | 12 (3.2) |
| No formal qualification | 5 (9.6) | 0 | 0 | 1 (1.6) | 0 | 0 | 0 | 0 | 0 | 0 | 0 | 0 | 5 (2.3) | | 1 (0.3) |
| Partner employment status | |  |  |  |  |  |  |  |  |  |  |  |  | |  |
| Working full time | 23  (54.8) | 19 (39.6) | 17 (65.4) | 40 (62.5) | 14 (66.7) | 32 (47.8) | 20 (71.4) | 32 (51.6) | 18 (78.3) | 33 (47.8) | 21 (91.3) | 49 (70.0) | 113 (69.3) | | 205 (53.9) |
| Working part time | 7  (16.7) | 11 (22.9) | 2  (7.7) | 9  (14.1) | 2  (9.5) | 11 (16.4) | 3  (10.7) | 2  (3.2) | 4  (17.4) | 7  (10.1) | 2  (8.7) | 8  (11.4) | 20 (12.3) | | 48  (12.6) |
| Student | 0 | 0 | 1 (3.8) | 0 | 0 | 0 | 1 (3.6) | 0 | 0 | 0 | 0 | 0 | 2 (1.2) | | 0 |
| Homemaker | 6  (14.3) | 4  (8.3) | 4  (15.4) | 1 (1.6) | 2  (9.5) | 3  (4.5) | 4  (14.3) | 5  (8.1) | 1  (4.3) | 3  (4.3) | 0 | 3  (4.3) | 17 (10.4) | | 19  (5.0) |
| Unemployed | 5 (11.9) | 3 (6.3) | 2 (7.7) | 2 (3.1) | 3 (14.3) | 3 (4.5) | 0 | 1 (1.6) | 0 | 0 | 0 | 1 (1.4) | 10 (6.1) | | 10 (2.6) |
| Prefer not to answer | 1 (2.4) | 0 | 0 | 0 | 0 | 0 | 0 | 2 (3.2) | 0 | 4 (5.8) | 0 | 0 | 1 (0.6) | | 6 (1.6) |
| Partner’s education* | |  |  |  |  |  |  |  |  |  |  |  |  | |  |
| 16 yr exams | 11  (26.2) | 12 (32.4) | 7  (26.9) | 25 (48.1) | 3  (14.3) | 9  (18.4) | 0 | 3  (7.1) | 1  (4.3) | 2  (4.3) | 0 | 0 | 22 (13.5) | | 51  (17.7) |
| Vocational qualification | 5  (11.9) | 4  (10.8) | 12 (46.2) | 23 (44.2) | 12 (54.5) | 25 (51.0) | 4  (14.3) | 0 | 1  (4.3) | 6  (12.8) | 2  (8.7) | 11 (18.0) | 35 (21.5) | | 69  (24.0) |
| Completed high school | 9  (21.4) | 6  (16.2) | 1 (3.8) | 1  (1.9) | 3  (14.3) | 6  (12.2) | 10 (35.7) | 17 (40.5) | 6  (26.1) | 18 (38.3) | 8 (34.8) | 16 (26.2) | 37 (22.7) | | 64  (22.2) |
| Under-graduate degree | 5  (11.9) | 9  (24.3) | 3  (11.5) | 1  (1.9) | 1  (4.8) | 2  (4.1) | 10 (35.7) | 14 (33.3) | 4  (17.4) | 3  (6.4) | 7 (30.4) | 15 (24.6) | 30 (18.4) | | 44  (15.3) |
| Post-graduate degree | 5  (11.9) | 4  (10.8) | 3  (11.5) | 2  (3.8) | 2 (9.5) | 7  (14.3) | 4  (14.3) | 7  (16.7) | 11 (47.8) | 17 (36.2) | 6 (26.1) | 17 (27.9) | 31 (19.0) | | 54  (18.8) |
| Other | 0 | 2 (5.4) | 0 | 0 | 1 (4.8) | 0 | 0 | 1 (2.4) | 0 | 1 (2.1) | 0 | 1 (1.4) | 1 (0.6) | | 5 (1.7) |
| No formal qualification | 7 (16.7) | 0 | 0 | 0 | 0 | 0 | 0 | 0 | 0 | 0 | 0 | 1 (1.4) | 7 (4.3) | | 1 (0.3) |
| Child’s age, mean (SD) | 14.4 (1.3) | 9.6 (1.9) | 14.9 (1.4) | 9.9 (1.6) | 14.4 (1.0) | 9.6 (1.9) | 14.4 (1.1) | 9.2  (1.9) | 14.5 (1.1) | 9.1 (2.2) | 14.6 (1.2) | 9.3 (2.1) | 14.5 (1.2) | | 9.5  (2.0) |
| Age at diagnosis, mean (SD) | 6.6  (3.9) | 5.3 (3.2) | 8.1 (3.2) | 5.9 (2.9) | 5.6 (3.8) | 5.7 (2.2) | 6.3 (4.2) | 4.3  (3.9) | 7.7 (4.8) | 3.0 (4.8) | 7.1 (4.1) | 4.1 (3.7) | 6.9  (4.1) | | 4.7  (3.7) |
| Time from first symptoms to diagnosis, mean yrs | 3.2 | 1.9 | 2.9 | 2.5 | 2 | 1.2 | 0.9 | 0.8 | 0.4 | 0.7 | 0.9 | 1 | 1.9 | | 1.3 |
| Time from diagnosis to medication, mean yrs | 0.8 | 0.4 | 0.6 | 0.3 | 0.9 | 0.8 | 0.3 | 0.4 | 0.2 | 0.5 | 0.3 | 0.4 | 0.5 | | 0.5 |
| Child had other therapy prior to starting medication | 18  (34.6) | 12 (25.0) | 11 (30.6) | 17 (26.6) | 19 (57.6) | 34 (50.7) | 9  (23.7) | 27 (43.5) | 12 (38.7) | 25 (36.2) | 13 (43.3) | 41 (58.6) | 82 (37.3) | | 156 (41.1) |
| Current medication | |  |  |  |  |  |  |  |  |  |  |  |  | |  |
| Methylphenidate IR (Ritalin) | 21  (40.4) | 24 (50.0) | 12 (33.3) | 39 (60.9) | 5  (15.2) | 24 (35.8) | 10 (26.3) | 21 (33.9) | 14 (45.2) | 23 (33.3) | 11 (36.7) | 24 (34.3) | 73 (33.2) | | 155 (40.8) |
| Methylphenidate long-acting | 23  (44.2) | 21 (43.8) | 20 (55.6) | 21 (32.8) | 24 (72.2) | 42 (62.7) | 12 (31.6) | 21 (33.9) | 13 (41.9) | 18 (26.1) | 6 (20.0) | 20 (28.6) | 98 (44.5) | | 143 (37.6) |
| Dextroamphetamine | 2  (3.8) | 1  (2.1) | 0 | 3 (4.7) | 0 | 0 | 6  (15.8) | 12 (19.4) | 4  (12.9) | 14 (20.3) | 5 (16.7) | 8  (11.4) | 17  (7.7) | | 38  (10.0) |
| Mixed amphetamine salts: IR | 3  (5.8) | 1  (2.1) | 0 | 0 | 0 | 0 | 5  (13.2) | 3  (4.8) | 5  (16.1) | 12 (17.4) | 0 | 5  (7.1) | 13  (5.9) | | 21  (5.5) |
| Mixed amphetamine salts: extended-release | 1  (1.9) | 1  (2.1) | 1  (2.8) | 0 | 0 | 0 | 4  (10.5) | 2  (3.2) | 3  (9.7) | 7  (10.1) | 3 (10.0) | 7  (10.0) | 12  (5.5) | | 17  (4.5) |
| Pemoline (Cylert) | 1  (1.9) | 2  (4.2) | 0 | 1  (1.6) | 0 | 0 | 2  (5.3) | 4  (6.5) | 7  (22.6) | 14 (20.3) | 1  (3.3) | 6  (8.6) | 11  (5.0) | | 27  (7.1) |
| Vyvanse | 1 (1.9) | 0 | 0 | 0 | 0 | 0 | 2 (5.3) | 3 (4.8) | 3 (9.7) | 9 (13.0) | 2 (6.7) | 2 (2.9) | 8 (3.6) | | 14 (3.7) |
| Atomoxetine (Strattera) | 7 (13.5) | 5 (10.4) | 1 (2.8) | 3 (4.7) | 7 (21.2) | 10 (14.9) | 1 (2.6) | 2 (3.2) | 2 (6.5) | 11 (15.9) | 2 (6.7) | 2 (2.9) | 20 (9.1) | | 33 (8.7) |
| Modafinil (Provigil) | 1 (1.9) | 2 (4.2) | 0 | 0 | 0 | 1 (1.5) | 2 (5.3) | 2 (3.2) | 5 (16.1) | 8 (11.6) | 2 (6.7) | 7 (10.0) | 10 (4.5) | | 20 (5.3) |
| Clonidine (Catapres) | 0 | 1 (2.1) | 1 (2.8) | 1 (1.6) | 1 (3.0) | 2 (3.0) | 4 (10.5) | 5 (8.1) | 5 (16.1) | 9 (13.0) | 0 | 4 (5.7) | 11 (5.0) | | 22 (5.8) |
| Guanfacine (Tenex) | 0 | 1 (2.1) | 0 | 0 | 0 | 0 | 3 (7.9) | 2 (3.2) | 2 (6.5) | 7 (10.1) | 1 (3.3) | 3 (4.3) | 6 (2.7) | | 13 (3.4) |
| Guanfacine extended-release (Intuniv) | 0 | 0 | 0 | 0 | 3 (9.1) | 2 (3.0) | 1 (2.6) | 0 | 0 | 0 | 2 (6.7) | 3 (4.3) | 6 (2.7) | | 5 (1.3) |
| Other medication | 3 (5.8) | 3 (6.3) | 4 (11.1) | 6 (9.4) | 0 | 0 | 1 (2.6) | 3 (4.8) | 1 (3.2) | 3 (4.3) | 0 | 1 (1.4) | 9 (4.1) | | 16 (4.2) |
| Child receives additional therapy currently | 30  (57.7) | 29 (60.4) | 19 (52.8) | 37 (57.8) | 20 (60.6) | 56 (83.6) | 24 (63.2) | 50  (80.6) | 23 (74.2) | 49  (71.0) | 22 (73.3) | 56 (80.0) | 138 (62.7) | | 277 (72.9) |
| Specify additional therapy | | |  |  |  |  |  |  |  |  |  |  |  | |  |
| Behavioral therapy (incl CBT) | 16  (30.8) | 16 (55.2) | 5  (13.9) | 16 (43.2) | 8  (24.2) | 34 (60.7) | 6  (15.8) | 17  (34.0) | 12 (38.7) | 26  (53.1) | 13 (43.3) | 30 (52.6) | 60 (27.3) | | 139 (50.2) |
| Individual counseling | 15  (28.8) | 9  (31.0) | 6  (16.7) | 10 (27.0) | 9  (27.3) | 25 (44.6) | 8  (21.1) | 17  (34.0) | 7  (22.6) | 21  (42.9) | 7  (23.3) | 26 (46.4) | 52 (23.6) | | 108 (39.0) |
| Family counseling and/or therapy | 8  (15.4) | 5  (17.2) | 4  (11.1) | 8  (21.6) | 7  (21.2) | 16 (28.6) | 5  (13.2) | 20  (40.0) | 6  (19.4) | 9  (18.4) | 6  (20.0) | 11 (19.6) | 36 (16.4) | | 69  (24.9) |
| Parent counseling and/or therapy | 4  (7.7) | 3  (10.3) | 2  (5.6) | 10 (27.0) | 4  (12.1) | 17 (30.4) | 2  (5.3) | 8  (16.0) | 2  (6.5) | 7  (14.3) | 1  (3.3) | 1  (1.8) | 15  (6.8) | | 46  (16.6) |
| Psycho-education | 8 (15.4) | 1 (3.4) | 1 (2.8) | 1 (2.7) | 1 (3.0) | 3 (5.4) | 1 (2.6) | 13 (26.0) | 1 (3.2) | 8 (16.3) | 1 (3.3) | 5 (8.9) | 13 (5.9) | | 31 (11.2) |
| Neuro-feedback | 2 (3.8) | 0 | 0 | 0 | 0 | 1 (1.8) | 2 (5.3) | 2 (4.0) | 1 (3.2) | 4 (8.2) | 0 | 0 | 5 (2.3) | | 7 (2.5) |
| Physical therapies | 5  (9.6) | 5  (17.2) | 3  (8.3) | 1  (2.7) | 7  (21.2) | 7  (12.5) | 6  (15.8) | 14  (28.0) | 8  (25.8) | 8  (16.3) | 2  (6.7) | 10 (17.9) | 31 (14.1) | | 45  (16.2) |
| Specific learning disability therapy | 12  (23.1) | 8  (17.6) | 4  (11.1) | 5  (13.5) | 3  (9.1) | 10 (17.9) | 3  (7.9) | 13  (26.0) | 4  (12.9) | 10  (20.4) | 1  (3.3) | 6  (10.7) | 27 (12.3) | | 52  (18.8) |
| Dietary supplements | 6 (11.5) | 3 (10.3) | 0 | 5 (13.5) | 0 | 3 (5.4) | 2 (5.3) | 6 (12.0) | 2 (6.5) | 2 (6.9) | 4 (13.3) | 7 (12.5) | 14 (6.4) | | 26 (9.4) |
| Dietary/nutrition changes | 7 (13.5) | 8 (27.6) | 1 (2.8) | 6 (16.2) | 0 | 5 (8.9) | 3 (7.9) | 3 (6.0) | 2 (6.5) | 6 (12.2) | 1 (3.3) | 3 (5.4) | 14 (6.4) | | 31 (11.2) |
| Other type of therapy | 1 (1.9) | 1 (3.4) | 1 (2.8) | 4 (10.8) | 2 (6.1) | 7 (12.5) | 0 | 0 | 0 | 1 (2.0) | 0 | 1 (1.8) | 4 (1.8) | | 14 (5.1) |
| Social group work | 0 | 5 (10.4) | 0 | - | 1 (3.0) | - | 0 | - | 0 | - | 0 | - | 1 (0.5) | | - |
| Pet therapy | 0 | 3 (60.0) | 0 | - | 1 (3.0) | - | 0 | - | 0 | - | 0 | - | 1 (0.5) | | - |
| Child psychology | 0 | 3 (60.0) | 1 (2.8) | - | 0 | - | 0 | - | 0 | - | 0 | - | 1 (0.5) | | - |
| Speech therapy | 1 (1.9) | 0 | 0 | - | 0 | - | 0 | - | 0 | - | 0 | - | 1 (0.5) | | - |
| Previous ADHD medication | 24  (46.2) | 5 (10.4) | 17 (47.2) | 18 (28.1) | 23 (69.7) | 22 (32.8) | 10 (26.3) | 14  (22.6) | 5  (16.1) | 15  (21.7) | 2  (6.7) | 13 (18.6) | 81 (36.8) | | 87  (22.9) |
| Methylphenidate IR (Ritalin) | 15 | 3  (60.0) | 13 | 14 (77.8) | 15 | 16 (72.7) | 5 | 5  (35.7) | 2 | 5  (33.3) | 1 | 3  (23.1) | 51 | | 46  (22.9) |
| Methylphenidate long-acting | 13 | 3  (60.0) | 1 | 3  (16.7) | 6 | 11 (50.0) | 2 | 3  (21.7) | 3 | 5  (33.3) | 0 | 3  (23.1) | 25 | | 27  (31.0) |
| Dextroamphetamine | 1 | 0 | 1 | 2 (11.1) | 0 | 0 | 3 | 3 (21.4) | 1 | 6 (40.0) | 0 | 4 (30.8) | 6 | | 15 (7.2) |
| Vyvanse | 0 | 0 | 0 | 0 | 1 | 1 (4.5) | 1 | 0 | 1 | 2 (13.3) | 0 | 0 | 3 | | 3 (3.4) |
| Atomoxetine (Strattera) | 4 | 0 | 2 | 2 (11.1) | 3 | 4 (18.2) | 3 | 1 (7.1) | 1 | 1 (6.7) | 0 | 0 | 13 | | 9 (10.3) |
| Other previous medication | 3 | 0 | 3 |  | 2 |  | 7 |  | 13 |  | 0 |  | 25 | |  |
| Reason for switching medication | | |  |  |  |  |  |  |  |  |  |  |  | |  |
| Prior treatment not effective | 17  (32.7) | 4  (80.0) | 9  (25.0) | 7  (38.9) | 9  (27.3) | 9  (40.9) | 7  (18.4) | 11  (78.6) | 1 | 11  (73.3) | 2  (6.7) | 11 (84.6) | 48 (21.8) | | 53  (60.9) |
| Other reason for switching | 6  (11.5) | 2  (40.0) | 7  (19.4) | 11 (61.1) | 14 (42.4) | 11 (50.0) | 2  (5.3) | 2  (14.3) | 1 | 1  (6.7) | 0 | 0 | 29 (13.2) | | 27  (31.0) |
| Prefer not to answer | 0 | 0 | 0 | 0 | 1 (3.0) | 1 (4.5) | 0 | 1 (7.1) | 1 | 3 (20.0) | 0 | 1 (7.7) | 2 (0.9) | | 6 (6.9) |
| Don't know why switched | 1 (1.9) | 0 | 1 (2.8) | 0 | 0 | 1 (4.5) | 2 (5.3) | 0 | 13 | 1 (6.7) | 0 | 1 (7.7) | 4 (1.8) | | 3 (3.4) |
| Aspect of prior treatment not effective | | |  |  |  |  |  |  |  |  |  |  |  | |  |
| Didn't last long enough | 8  (15.4) | 3  (75.0) | 4  (11.1) | 5  (71.4) | 5  (15.2) | 5  (55.6) | 3  (7.9) | 5  (45.5) | 3  (9.7) | 5  (45.5) | 1  (3.3) | 5  (45.5) | 24 (10.9) | | 28  (52.8) |
| Wasn't optimal | 9 (17.3) | 1 (25.0) | 2 (5.6) | 5 (71.4) | 6 (18.2) | 7 (77.8) | 4 (10.5) | 4 (36.4) | 2 (6.5) | 8 (72.7) | 2 (6.7) | 8 (72.7) | 25 (11.4) | | 33 (62.3) |
| Caused side effects | 5 (9.6) | 3 (75.0) | 3 (8.3) | 2 (28.6) | 2 (6.1) | 2 (22.2) | 2 (5.3) | 1 (9.1) | 0 | 1 (9.1) | 1 (3.3) | 1 (9.1) | 13 (5.9) | | 10 (18.9) |
| Didn't want treatment effect beyond school time | 2 (3.8) | 0 | 0 | 0 | 1 (3.0) | 0 | 0 | 1 (9.1) | 0 | 3 (27.3) | 0 | 3 (27.3) | 3 (1.4) | | 7 (13.2) |
| Interaction with another medication | 1 (1.9) | 0 | 0 | 0 | 0 | 0 | 0 | 2 (18.2) | 0 | 1 (9.1) | 0 | 1 (9.1) | 1 (0.5) | | 4 (7.5) |
| Other aspect of treatment not effective | 2 (3.8) | 0 | 0 | 0 | 0 | 0 | 0 | 1 (9.1) | 0 | 1 (9.1) | 0 | 2 (18.2) | 2 (0.9) | | 1 (1.9) |
| Concerns about current medication | 15  (28.8) | 15 (31.3) | 14 (38.9) | 26 (41.3) | 18 (54.5) | 35 (52.2) | 13 (34.2) | 28  (45.9) | 7  (22.6) | 24  (34.8) | 20 (66.7) | 52 (74.3) | 87 (39.5) | | 180 (47.6) |
| Side effect concerns | 7  (7.7) | 5  (33.3) | 7  (19.4) | 8  (29.6) | 10 (30.3) | 28 (77.8) | 8  (21.1) | 13  (46.4) | 3  (9.7) | 8  (33.3) | 10 (33.3) | 25 (48.1) | 41 (18.6) | | 87  (47.8) |
| Take too many times/day | 2  (3.8) | 1  (2.1) | 2  (5.6) | 7  (10.9) | 1  (3.0) | 4  (6.0) | 4  (10.5) | 5  (8.1) | 0 | 8  (11.6) | 5  (16.7) | 12 (17.1) | 14  (6.4) | | 37  (9.7) |
| Doesn’t last long enough | 7 (13.5) | 6 (40.0) | 1 (2.8) | 6 (21.4) | 5 (15.2) | 7 (20.0) | 0 | 2 (6.9) | 1 (3.2) | 3 (12.5) | 3 (10.0) | 8 (15.4) | 17 (7.7) | | 32 (17.5) |
| Not easy to use | 0 | 4 (26.7) | 0 | 4 (14.3) | 1 (3.0) | 2 (5.7) | 1 (2.6) | 3 (10.3) | 1 (3.2) | 3 (12.5) | 4 (13.3) | 5 (9.6) | 7 (3.2) | | 21 (11.5) |
| Doesn't want treatment effect beyond school time | 2  (3.8) | 2  (13.3) | 1  (2.8) | 3  (10.7) | 3  (9.1) | 5  (14.3) | 3  (7.9) | 8  (27.6) | 1  (3.2) | 7  (29.2) | 2  (6.7) | 12 (23.1) | 12  (5.5) | | 37  (20.2) |
| Prefer not to answer | 0 | 0 | 1 (2.8) | 1 (3.6) | 0 | 1 (2.9) | 0 | 0 | 1 (3.2) | 1 (4.2) | 0 | 3 (5.8) | 2 (0.9) | | 6 (3.3) |
| Don't know why concerned | 0 | 0 | 0 | 2 (7.1) | 1 (3.0) | 0 | 0 | 1 (3.4) | 0 | 1 (4.2) | 1 (3.3) | 1 (1.9) | 2 (0.9) | | 5 (2.7) |
| Incompatible with other medication | 0 | – | 0 | – | 0 | – | 1 (2.6) | – | 0 | – | 0 | – | 1 (0.5) | | – |
| Increased problems on medication | 0 | – | 1 (2.8) | – | 0 | – | 0 | – | 0 | – | 0 | – | 1 (0.5) | | – |
| Dependence | 0 | – | 1 (2.8) | – | 1 (3.0) | – | 0 | – | 0 | – | 0 | – | 2 (0.9) | | – |
| Non-adherence | 1 (1.9) | – | 0 | – | 0 | – | 0 | – | 0 | – | 0 | – | 1 (0.5) | | – |
| Long-term health effects | 4 (7.7) | – | 0 | – | 3 (9.1) | – | 1 (2.6) | – | 0 | – | 1 (3.3) | – | 9 (4.1) | | – |
| Alternative drugs available | 0 | – | 0 | – | 0 | – | 0 | – | 1 (3.2) | – | 0 | – | 1 (0.5) | | – |
| Costs | 0 | – | 1 (2.8) | – | 0 | – | 0 | – | 0 | – | 0 | – | 1 (0.5) | | – |
| Aware of abuse potentia | 35  (67.3) | N/A | 29 (80.6) | N/A | 25 (75.8) | N/A | 25 (65.8) | N/A | 15 (48.4) | N/A | 17 (56.7) | N/A | 146 (66.4) | | N/A |
| Ever been concerned about your child abusing his/her medication | 6  (11.5) | N/A | 6  (16.7) | N/A | 4  (12.1) | N/A | 18 (47.4) | N/A | 12 (38.7) | N/A | 18 (60.0) | N/A | 64 (29.1) | | N/A |

*Missing data

IR, immediate release; N/A, not applicable
